# Factors related to rapid progression of non‐small cell lung cancer in Chinese patients treated using single‐agent immune checkpoint inhibitor treatment

**DOI:** 10.1111/1759-7714.13370

**Published:** 2020-03-05

**Authors:** Liang Zhang, Lianwei Bai, Xianhong Liu, Ying Liu, Shuang Li, Jingjing Liu, Shuang Zhang, Changliang Yang, Xiubao Ren, Ying Cheng

**Affiliations:** ^1^ Tianjin Medical University Cancer Institute and Hospital, National Clinical Research Center for Cancer; Key Laboratory of Cancer Prevention and Therapy; Tianjin's Clinical Research Center for Cancer; Key Laboratory of Cancer Immunology and Biotherapy Tianjin China; ^2^ Division of Thoracic Oncology Jilin Cancer Hospital Changchun China; ^3^ The Third Division of Medical Oncology Jilin Cancer Hospital Changchun China

**Keywords:** Immune checkpoint inhibitor, non‐small cell lung cancer, rapid progression

## Abstract

**Background:**

Immune checkpoint inhibitors (ICIs) have revolutionized the treatment of non‐small cell lung cancer (NSCLC). While rapid progression (RP) has been proposed as a non‐negligible pattern of response to ICIs, its definition and related factors remain unclear. This study aimed to develop a clinical definition of RP and to identify related factors.

**Methods:**

We retrospectively evaluated Chinese patients who had received an ICI as second‐line or later treatment for locally advanced or metastatic NSCLC at a single center. We defined RP as radiological progression at the first response assessment (<2 months after starting the ICI), as well as confirmation of progressive disease or cancer‐related death occurring at <3 months. The clinical outcomes were compared for patients with RP or non‐RP to identify prognostic factors.

**Results:**

The study evaluated 74 eligible patients with detailed records regarding their ICI therapy, including 25 patients (33.8%) who had experienced RP. Relative to patients with non‐RP, patients with RP had significantly shorter median progression‐free survival (1.7 months [95% CI: 1.4–2.0 months] vs. 6.3 months [95% CI 5.2–7.3 months], *P* < 0.001; hazard ratio: 0.14, 95% CI: 0.08–0.25) and significantly shorter median overall survival (8.2 months [95% CI 3.0–13.4 months] vs. 22.6 months [95% CI 17.0–28.1 months], *P* < 0.001; hazard ratio: 0.27, 95% CI: 0.15–0.49). Multivariate analysis revealed that RP was independently predicted by the presence of ≥3 metastatic sites (*P* = 0.039) and a neutrophil‐to‐lymphocyte ratio of ≥3 (*P* = 0.044).

**Conclusions:**

Among NSCLC patients, RP was a common response to ICI monotherapy and was associated with dramatically reduced progression‐free and overall survival. Care is needed when selecting ICI monotherapy for these patients, especially if they have ≥3 metastatic sites or a neutrophil‐to‐lymphocyte ratio of ≥3.

**Key points:**

Significant findings of the study: Patients with rapid progression after immune checkpoint inhibitor monotherapy had poor survival outcomes. The number of metastatic sites and the neutrophil‐to‐lymphocyte ratio may independently predict treatment response in this setting.What this study adds: This is the first study to evaluate rapid progression after second‐line or later single‐agent immunotherapy in a Chinese population. Our findings may help establish effective immunotherapy strategies for NSCLC.

## Introduction

In the era of immuno‐oncology, inhibitors of programmed cell death 1 (PD‐1) and programmed cell death ligand 1 (PD‐L1) have provided robust survival benefits for patients with locally advanced and metastatic non‐small cell lung cancer (NSCLC). Single‐agent nivolumab or atezolizumab treatments are now standard second‐line treatments, regardless of PD‐L1 status, as well as pembrolizumab for patients with a PD‐L1 tumor proportion score of ≥1%.^1^, ^2^, ^3^, ^4^ Pembrolizumab monotherapy is also approved as first‐line treatment for advanced NSCLC in patients with PD‐L1 expression of >50%, and this indication was recently extended to patients with PD‐L1 expression of ≥1%.^5^, ^6^

However, most NSCLC patients who receive single‐agent immune checkpoint inhibitor (ICI) treatment do not experience antitumor activity in the second or later lines, with some patients even exhibiting a response pattern involving accelerated or rapid progression (RP). This phenomenon of accelerating tumor growth was also observed based on the early intersection of the progression‐free survival (PFS) and overall survival (OS) curves with the initially inferior outcomes from several phase III trials of ICIs versus docetaxel for relapsed NSCLC.^2^, ^3^, ^4^ Hyperprogressive disease (HPD) resulting from immunotherapy has also been reported as a special response pattern.^7^, ^8^, ^9^ Nevertheless, the evaluation of HPD relies on the tumor growth rate or tumor growth kinetics, which requires multiple computed tomography (CT) scans. Thus, it can be complicated and inconvenient to apply this method of evaluating HPD in clinical practice. Furthermore, HPD does not include all patients who do not benefit from ICIs. Thus, RP has been suggested as a new potential alternative response pattern, although its definition and related factors remain unclear. This study aimed to develop a clinical definition of RP and to identify risk factors associated with this entity.

## Methods

### Study population

We retrospectively reviewed all patients who were hospitalized at Jilin Cancer Hospital between 1 March 2016 and 30 March 2019. The inclusion criteria were: a histologically confirmed diagnosis of NSCLC; stage IIIB or IV disease according to the American Joint Committee on Cancer guidelines (version 7); measurable disease according to version 1.1 of the Response Evaluation Criteria in Solid Tumors (RECIST v1.1); Eastern Cooperative Oncology Group (ECOG) performance status of ≤2; patients harboring EGFR mutations or ALK rearrangement were required to have previously received tyrosine kinase inhibitor (TKI) therapy and experienced disease progression; second‐line or later ICI monotherapy (nivolumab, pembrolizumab, or atezolizumab); confirmation of response to the ICI; adequate organ function; and available clinical data. The key exclusion criteria were: no confirmation of treatment response and significant comorbidities or concurrent diseases that might influence the continuous administration of ICIs or lead to fatal clinical outcomes, such as active autoimmune disease, systemic immunosuppression, or unstable symptoms of brain metastasis.

Eligible patients received intravenous nivolumab at a dose of 3 mg/kg every two weeks, pembrolizumab at a fixed dose of 200 mg every three weeks, or atezolizumab at a fixed dose of 1200 mg every three weeks. Treatment continued until disease progression, discontinuation because of toxicity, or other reasons. Patients who received pembrolizumab required a PD‐L1 tumor proportion score of ≥1%, while patients were allowed to receive nivolumab or atezolizumab regardless of PD‐L1 expression. The ICIs could be continued after the initial determination of PD until the physician determined that the treatment provided no clinical benefit.

The study was conducted in accordance with good clinical practice guidelines and the declaration of Helsinki. The requirement for informed consent was waived based on the retrospective design.

### Clinicopathological and laboratory data

The clinicopathological and laboratory data of patients were collected from their medical records. The present study considered age, sex, ECOG PS, smoking history, disease stage before ICI therapy, histological type, EGFR mutation or ALK rearrangement status, number and location of metastatic sites (lungs, liver, brain, bone, or adrenal gland), previous thoracic radiotherapy, previous therapy lines, tumor burden (measured as the sum of the unidimensional diameters of up to five target lesions per RECIST v1.1), PD‐L1 expression, time since the last treatment, antibiotic use <2 months before starting ICIs, corticosteroid use <30 days before starting ICIs (equivalent to ≥10 mg of prednisone), the neutrophil‐to‐lymphocyte ratio (NLR) in peripheral blood, and serum lactate dehydrogenase (LDH) levels.

The response to ICI therapy was assessed based on RECIST v1.1, with responses defined as complete response (CR), partial response (PR), stable disease (SD), or progressive disease (PD). Response evaluations were generally performed every 6–8 weeks after starting ICI therapy, and involved imaging confirmation. The PFS interval was defined as the time from starting ICI treatment to the first instance of disease progression or death because of any cause. The OS interval was defined as the time from starting ICI treatment to death because of any cause.

We considered RP present if there was radiological evidence of progression at the first response assessment (<two months after starting ICI treatment), with confirmation of PD occurrence or death because of disease progression within three months. Patients who did not fulfill the RP criteria were assigned to the nonrapid progression group (non‐RP).

### Statistical analysis

The RP and non‐RP groups were compared to identify relevant prognostic factors. Univariable and multivariable Cox proportional hazard regression models were used to identify prognostic factors, and the results were reported as hazard ratios (HRs) with 95% confidence intervals (CIs). The OS and PFS curves were estimated using the Kaplan‐Meier method and compared using the log‐rank test. All reported *P*‐values were two‐sided, and *P*‐values of <0.05 were considered statistically significant. All statistical analyses were performed using IBM SPSS software (version 25.0; IBM Corp., Armonk, New York).

## Results

### Patient characteristics

We identified 74 eligible patients who received single‐agent ICI therapy as second‐line or later treatment for locally advanced or metastatic NSCLC (Table 1).

**Table 1:** Baseline clinical and pathological characteristics

	All patients *N* = 74	Rapid progression *N* = 25	Nonrapid progression *N* = 49
Age (years)			
Median (range)	62 (38–78)	60 (38–71)	62 (45–78)
≥ 65 years	21(28.4%)	5 (20.0%)	16 (32.7%)
< 65 years	53(71.6%)	20 (80.0%)	33 (67.3%)
Sex			
Male	56 (75.7%)	20 (80.0%)	36 (73.5%)
Female	18 (24.3%)	5 (20.0%)	13 (26.5%)
Smoking history			
Never	31 (41.9%)	12 (48.0%)	19 (38.8%)
Current or former	43 (58.1%)	13 (52.0%)	30 (61.2%)
Stage			
IIIB	14 (18.9%)	4 (16.0%)	10 (20.4%)
IV	60 (81.1%)	21 (84.0%)	39 (79.6%)
Histology			
Adenocarcinoma	54 (73.0%)	17 (68.0%)	37 (75.5%)
Squamous	20 (27.0%)	8 (32.0%)	12 (24.5%)
ECOG PS			
0–1	63 (85.1%)	18 (72.0%)	45 (91.8%)
2	11 (14.9%)	7 (28.0%)	4 (8.2%)
*EGFR/ALK* mutational status			
Positive	12 (16.2%)	7 (28.0%)	5 (10.2%)
Negative	42 (56.8%)	10 (40.0%)	32 (65.3%)
Missing	20 (27.0%)	8 (32.0%)	12 (24.5%)
Metastatic site			
Liver	10 (13.5%)	4 (16.0%)	6 (12.2%)
Brain	9 (12.2%)	4 (16.0%)	5 (10.2%)
Bone	24 (32.4%)	8 (32.0%)	16 (32.7%)
Adrenal gland	9 (12.2%)	4 (16.0%)	5 (10.2%)
Lung	37 (50.0%)	12 (48.0%)	25 (51.0%)
Number of metastatic sites			
0–2	45 (60.8%)	11 (44.0%)	34 (69.4%)
≥ 3	29 (39.2%)	14 (56.0%)	15 (30.6%)
Tumor burden			
< 10 cm	59 (79.7%)	19 (76.0%)	40 (81.6%)
≥ 10 cm	15 (20.3%)	6 (24.0%)	9 (18.4%)
Thoracic radiotherapy			
Yes	16 (21.6%)	5 (20.0%)	11 (22.4%)
No	58 (78.4%)	20 (80.0%)	38 (77.6%)
Previous therapy lines			
1	49 (66.2%)	14 (56.0%)	35 (71.4%)
≥ 2	25 (33.8%)	11 (44.0%)	14 (28.6%)
ICIs			
Nivolumab	50 (67.6%)	14 (56.0%)	36 (73.5%)
Pembrolizumab	12 (16.2%)	7 (28.0%)	5 (10.2%)
Atezolizumab	12 (16.2%)	4 (16.0%)	8 (16.3%)
PD‐L1 expression			
≥ 1%	23(31.1%)	7 (28.0%)	16 (32.6%)
< 1%	16 (21.6%)	9 (36.0%)	7 (14.3%)
Missing	35 (47.3%)	9 (36.0%)	26 (53.1%)
Receiving antibiotics < 2 months before ICIs			
Yes	12 (16.2%)	3 (12.0%)	9 (18.4%)
No	62 (83.8%)	22 (88.0%)	40 (81.6%)
Receiving corticosteroids < 30 days before ICIs			
Yes	12 (16.2%)	3 (12.0%)	9 (18.4%)
No	62 (83.8%)	22 (88.0%)	40 (81.6%)
Time since the last treatment			
< 6 months	57 (77.0%)	18 (72.0%)	39 (79.6%)
≥ 6 months	17 (23.0%)	7 (28.0%)	10 (20.4%)
NLR			
≥ 3	42 (56.8%)	19 (76.0%)	23 (46.9%)
< 3	32 (43.2%)	6 (24.0%)	26 (53.1%)
LDH			
Normal	57 (77.0%)	18 (72.0%)	39 (79.6%)
Elevated	17 (23.0%)	7 (28.0%)	10 (20.4%)

ALK, anaplastic lymphoma kinase; ECOG PS, Eastern Cooperative Oncology Group performance status; EGFR, epidermal growth factor receptor; ICIs, immune checkpoint inhibitors; LDH, lactate dehydrogenase; NLR, neutrophil‐to‐lymphocyte ratio; PD‐L1, programmed cell death ligand 1.

The ICI treatments involved nivolumab (50 patients, 67.6%), pembrolizumab (12 patients, 16.2%), and atezolizumab (12 patients, 16.2%). Immunohistochemistry‐based testing for PD‐L1 expression was performed for 39 patients, which revealed positive results (≥1% staining among the tumor cells) in 23 patients. All patients with adenocarcinoma underwent genetic testing for EGFR/ALK abnormalities, although this was not routinely performed for squamous cell carcinoma.

Based on our RP definition, we assigned 25 patients to the RP group (33.8%) and 49 patients to the non‐RP group (66.2%).

### Clinical outcomes

The median follow‐up after starting ICI treatment was 14.1 months (95% CI: 1.7–39.3 months). At the time of the analysis, 64 patients (86.5%) had imaging‐confirmed PD, and 44 patients had died (59.5%). The median overall PFS was 4.2 months (95% CI: 2.6–5.8 months) and the median overall OS was 16.8 months (95% CI: 12.5–21.0 months). The objective response rate (ORR) was 28.4% and the median duration of response (DOR) was 18.6 months (95% CI: 16.4–20.8 months).

**Figure 1:**
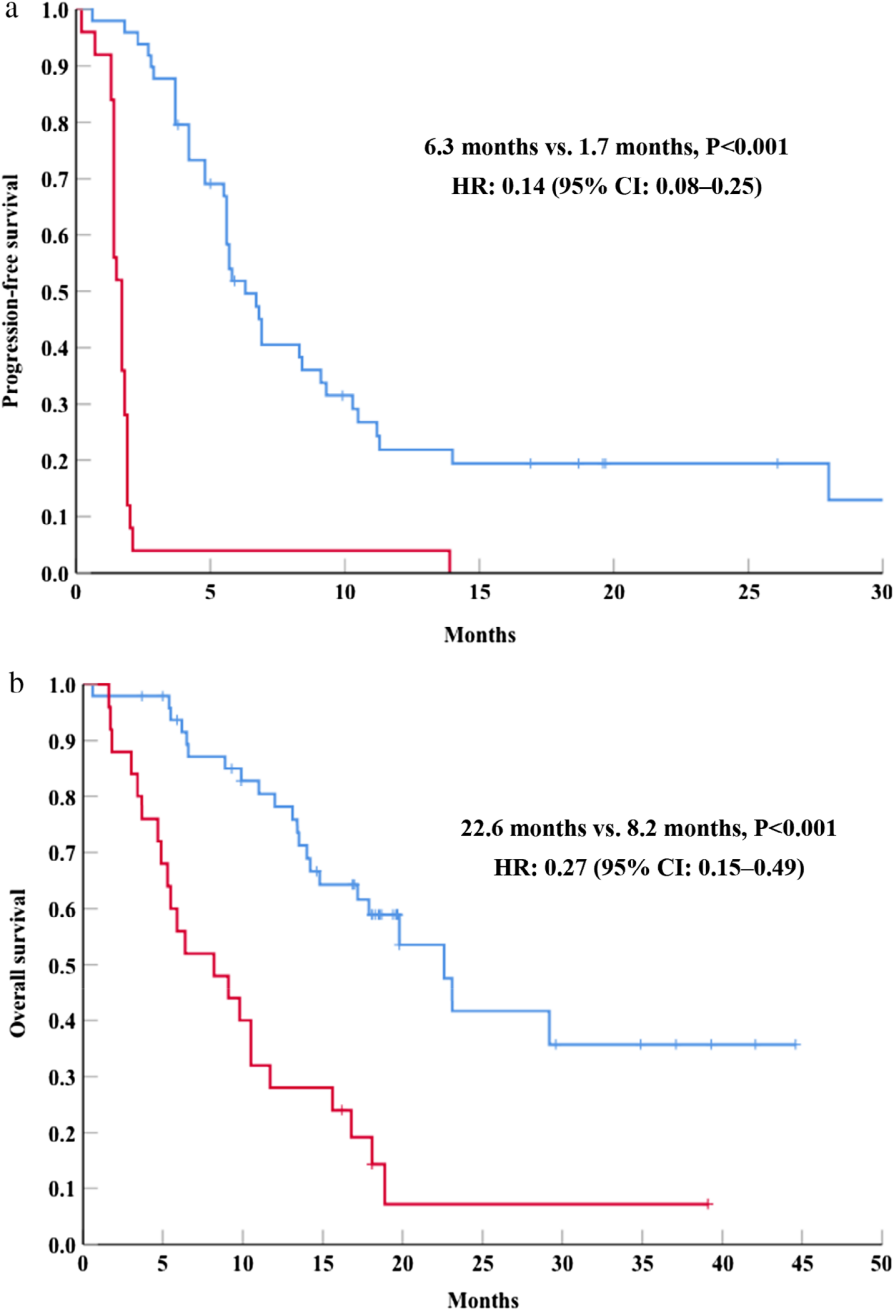
Kaplan‐Meier curves for (**a**) progression‐free‐survival and (**b**) overall survival in patients with or without rapid progression. HR, hazard ratio; CI, confidence interval. (**a**) (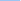) Non‐rapid progression and (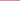) rapid progression. (**b**) (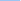) Non‐rapid progression and (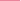) rapid progression.

We identified PD in 25 patients (100%) from the RP group and in 39 patients (79.6%) from the non‐RP group. All eligible patients were evaluable for OS, with death observed for 22 patients (88.0%) in the RP group and for 22 patients (44.9%) in the non‐RP group. The median PFS was 1.7 months in the RP group (95% CI: 1.4–2.0 months) and 6.3 months in the non‐RP group (95% CI: 5.2–7.3 months, *P* < 0.001). The non‐RP group had significantly better PFS (HR: 0.14, 95% CI 0.08–0.25) (Fig 1a). The median OS was 8.2 months in the RP group (95% CI: 3.0–13.4 months) and 22.6 months in the non‐RP group (95% CI: 17.0–28.1 months, *P* < 0.001). The non‐RP group had significantly better OS (HR: 0.27, 95% CI: 0.15–0.49) (Fig 1b). Thus, the RP group experienced significantly poorer response to single‐agent ICI treatment in terms of PFS and OS.

### Potential predictors of rapid progression

Univariate and multivariate Cox regression analyses were performed to identify clinicopathological and laboratory factors that were associated with RP and survival (Table 2). The univariate analyses revealed that RP was not significantly associated with age, sex, smoking history, disease stage, histological type, metastatic site location, tumor burden, PD‐L1 expression status, previous thoracic radiotherapy, previous therapy line, time since the last treatment, antibiotic or corticosteroid use before starting ICI treatment, or serum LDH levels. However, we found that RP was significantly associated with an ECOG PS of 2, genetic EGFR/ALK alterations, ≥3 metastatic sites, and a NLR of ≥3. These significant factors were included in the multivariate analyses.

**Table 2:** Univariate and multivariate analyses of factors associated with rapid progression

	Univariate analysis		Multivariate analysis	
	HR (95% CI)	*P*‐value	HR (95% CI)	*P*‐value
Age (< 65 vs. ≥ 65 years)	0.52 (0.16–1.62)	0.258	–	–	
Sex (male vs. female)	0.69 (0.22–2.23)	0.537	–	–	
Smoking (never vs. current/former)	1.46 (0.55–3.86)	0.448	–	–	
Stage (IIIB vs. IV)	0.74 (0.21–2.66)	0.648	–	–	
Histology (adenocarcinoma vs. squamous)	0.69 (0.24–2.00)	0.493	–	–	
ECOG PS (0–1 vs. 2)	0.23 (0.06–0.88)	0.031	0.98 (0.54–2.47)	0.213	
*EGFR/ALK* mutation (No vs. Yes)	0.22 (0.06–0.86)	0.029	0.80 (0.45–1.62)	0.094	
Liver metastases (No vs. Yes)	0.73 (0.19–2.88)	0.656	–	–	
Brain metastases (No vs. Yes)	0.60 (0.15–2.45)	0.474	–	–	
Bone metastases (No vs. Yes)	1.03 (0.37–2.89)	0.955	–	–	
Adrenal gland metastases (No vs. Yes)	0.60 (0.15–2.45)	0.474	–	–	
Lung metastases (No vs. Yes)	1.13 (0.43–2.96)	0.806	–	–	
Metastatic sites (< 3 vs. ≥ 3)	0.35 (0.13–0.94)	0.037	0.59 (0.46–0.78)	0.039	
Tumor burden (< 10 vs. ≥ 10 cm)	0.71 (0.22–2.29)	0.570	–	–	
PD‐L1 expression (positive vs. negative)	0.34 (0.09–1.28)	0.112	–	–	
Previous therapy lines (1 vs. ≥ 2)	0.49 (0.18–1.34)	0.165	–	–	
Receiving antibiotics (No vs. yes)	0.61 (0.15–2.47)	0.485	–	–	
Receiving corticosteroid (No vs. yes)	0.61 (0.15–2.47)	0.485	–	–	
Time since the last treatment (< 6 vs. ≥ 6 months)	0.66 (0.22–2.01)	0.464	–	–	
NLR (< 3 vs. ≥ 3)	0.28 (0.10–0.82)	0.020	0.34 (0.12–0.97)	0.044	
LDH (normal vs. elevated)	0.66 (0.22–2.01)	0.464	–	–	

ALK, anaplastic lymphoma kinase; CI, confidence interval; ECOG PS, Eastern Cooperative Oncology Group performance status; EGFR, epidermal growth factor receptor; HR, hazard ratio; LDH, lactate dehydrogenase; NLR, neutrophil‐to‐lymphocyte ratio; PD‐L1, programmed cell death ligand 1.

**Figure 2:**
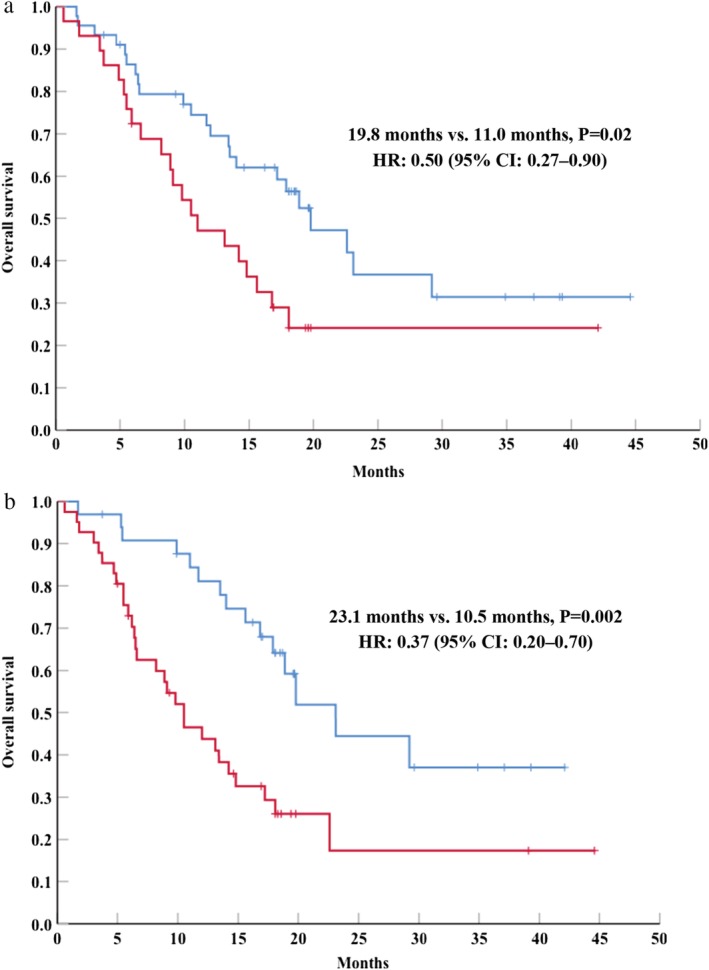
Kaplan‐Meier curves for overall survival according to the number of metastatic sites (**a**) or the NLR (**b**). HR, hazard ratio; CI, confidence interval; NLR, neutrophil‐to‐lymphocyte ratio. (**a**) (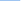) <3 Metastatic sites and (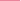) ≥3 metastatic sites. (**b**) (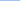) NLR of <3 and (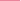) NLR of ≥3.

The multivariate analyses revealed that RP was independently predicted by the patient having ≥3 metastatic sites (*P* = 0.039) and a NLR of ≥3 (*P* = 0.044), although no independent relationships were observed with an ECOG PS of 2 or genetic EGFR/ALK alterations. We observed a significant difference in OS between patients with <3 metastatic sites (19.8 months, 95% CI: 14.1–25.5 months) and patients with ≥3 metastatic sites (11.0 months, 95% CI: 5.9–16.1 months, *P* = 0.02). Fewer metastatic sites were associated with significantly better OS (HR: 0.50, 95% CI: 0.27–0.90) (Fig 2a). We also observed a significant difference in OS between patients with a NLR of <3 (23.1 months, 95% CI: 16.7–29.5 months) and patients with a NLR of ≥3 (10.5 months, 95%CI: 6.9–14.1 months, *P* = 0.002). A lower NLR was associated with significantly better OS (HR: 0.37, 95% CI: 0.20–0.70) (Fig 2b).

## Discussion

Single‐agent ICI therapy can lead to a unique response pattern involving accelerated tumor growth. For example, in the study by Ferrara *et al*. the HPD rates were 13.8% for immunotherapy and 5.1% for chemotherapy as second‐line or later treatment for NSCLC.^9^ However, the identification of HPD based on the tumor growth rate or tumor growth kinetics requires at least two CT scans from before ICI therapy and one CT scan during ICI treatment, which may be difficult to implement in routine clinical practice. In addition, the definition of HPD does not include patients who experience an early death and does not represent the entire population of patients who never benefit from immunotherapy. A combined analysis of the CheckMate017 and CheckMate057 studies revealed that nivolumab treatment was associated with four‐year OS rates of 58% for CR/PR cases, 19% for SD cases, and 4% for PD cases, relative to a four‐year OS rate of 5% for docetaxel treatment.^10^ This suggests that the OS after starting immunotherapy is closely related to the RECIST v1.1‐based response, and immunotherapy is associated with a lower four‐year OS rate than docetaxel in patients with PD. Thus, the presence of RP, based on the RECIST v1.1 response or early death, might be a more appropriate alternative indicator for use in clinical practice. Furthermore, PD was confirmed by imaging to exclude patients with pseudo‐progression, which is another pattern of response to ICI therapy.

In the present study, the incidence of RP was 33.8% among NSCLC patients treated using second‐line or later single‐agent ICI therapy. Furthermore, relative to patients with non‐RP, patients with RP experienced significantly shorter PFS and OS, which suggests that it is important to consider related risk factors before starting ICI therapy. Our findings suggest that the number of metastatic sites at baseline was associated with RP, which is consistent with a previous report that this factor is related to HPD.^9^ Our study also showed that RP was not associated with tumor burden, which is probably because the RECIST v1.1‐defined target lesions do not always truly reflect the entire tumor burden, especially in patients with nonmeasurable disease, such as bone metastases, pleural effusion, or malignant atelectasis. Several studies have also showed that patients with high tumor burden experience poor OS after starting immunotherapy, which suggests that tumor burden may be a prognostic factor rather than a predictor of response.^9^, ^11^

Another study has suggested that an age of >65 years was associated with hyperprogression,^7^ although conflicting results were observed in the present study and several other studies.^8^, ^9^ Thus, we speculate that older age may not be a factor for excluding patients from receiving ICI therapy. Furthermore, we observed that RP was not associated with other baseline factors, including sex, disease stage, histological type, location of metastatic sites, thoracic radiotherapy, and time since the last treatment. Interestingly, we observed that an ECOG PS of two was associated with RP, but this association disappeared in the multivariate analysis. Thus, it remains unclear whether patients with a poor PS should receive ICI treatment, especially for patients who would not qualify for clinical trials.

Corticosteroids are commonly used to treat various complications in patients with advanced NSCLC. However, these drugs have immunosuppressive properties and may influence T cell function, which suggests that they may decrease the efficacy of immunotherapy.^12^ Moreover, baseline corticosteroid use equivalent to ≥10 mg of prednisone is associated with decreased ORR, PFS, and OS after starting ICI therapy.^13^ The gut microbiome was also recently shown to have direct effects on the innate and adaptive immune responses,^14^ with broad‐spectrum antibiotic treatment adversely influencing the outcomes of ICI therapy through the modulation of the intestinal microbiota.^15^, ^16^ A prospective study of 196 patients with NSCLC also concluded that antibiotic use before starting ICI therapy was associated with poorer rates of treatment response and survival.^17^ However, our findings suggest that corticosteroid or antibiotic use before ICI treatment was not related to RP. Thus, corticosteroids and antibiotics may potentially be useful if the need is urgent before starting ICI therapy, although further studies are needed regarding this subject.

The inflammation process may help explain immunoresistance in patients with cancer.^18^ The peripheral blood NLR and LDH levels may be potential inflammatory biomarkers in patients with cancer, and elevated values in several cancer types are associated with poor outcomes.^19^, ^20^, ^21^, ^22^ Our study indicated that a NLR of ≥3 independently predicted RP and was associated with poor survival, although LDH levels were not associated with RP or survival. Thus, the NLR may be a readily available prognostic factor for patients receiving ICIs. However, the NLR is a dynamic marker, and further studies are needed to determine whether changes in NLR during ICI treatment can help predict outcomes.

Approximately 50% of Chinese patients with adenocarcinoma have *EGFR* mutations, and this rate is significantly higher than among Caucasians.^23^ In this context, the use of ICIs remains controversial for patients harboring *EGFR* mutation and resistance to TKIs. Several studies have suggested that tumors with genetic *EGFR/ALK* alterations respond less well to ICI therapy than tumors without genetic *EGFR/ALK* alterations.^24^, ^25^, ^26^ The present study revealed that genetic *EGFR/ALK* alterations were associated with RP in the univariate analysis, although this association disappeared in the multivariate analysis. Nevertheless, the lack of an independent association could be related to the small size of this patient subgroup. A previous study has revealed an inverse relationship between *EGFR* mutation and PD‐L1 expression,^27^ with *EGFR* mutations being associated with a lack of T cell infiltration and noninflammatory tumor microenvironment.^28^ These features may explain poor outcomes after starting immunotherapy for patients with *EGFR* mutations. For example, the ATLANTIC study evaluated durvalumab in patients harboring *EGFR/ALK* mutations with TKI resistance, and revealed ORRs of 12.2% at PD‐L1 expression of ≥25% and 3.6% at PD‐L1 expression of <25%.^29^ Thus, the clinical activity of durvalumab was encouraging in *EGFR*‐mutated tumors with PD‐L1 expression of ≥25%. However, several phase III studies have revealed inconsistent relationships between PD‐L1 expression level and the response to second‐line ICI treatment.^1^, ^2^, ^3^, ^4^ Our results also indicated that there was no significant relationship between PD‐L1 expression and RP. We did not include the tumor mutational burden in the present study, because it is complex to evaluate and not routinely used in clinical practice. While a previous study has suggested that the tumor mutational burden may be associated with smoking,^30^ we found that smoking history did not predict RP in the present study.

The present study also has some important limitations. First, the retrospective single‐center design and small sample size are associated with risks of bias. Second, further confirmation is needed regarding whether our definition of RP is feasible and practical. Third, additional studies are needed to further identify and validate factors that can predict the response to ICI treatment in patients with NSCLC.

In conclusion, we found that RP was a common response pattern to second‐line or later single‐agent ICI treatment for NSCLC, and that RP was associated with significantly decreased PFS and OS. These findings may help guide the use of immunotherapy for NSCLC in routine clinical practice. For example, care is needed when selecting ICI monotherapy for NSCLC patients with risk factors for RP, and further studies are needed to better understand the mechanism of RP in patients with NSCLC.

## Disclosure

The authors declare that there are no conflicts of interest.
